# Influencing Pain Inferences Using Random Numerical Anchoring: Randomized Controlled Trial

**DOI:** 10.2196/17533

**Published:** 2020-03-09

**Authors:** Rebecca Elizabeth Lewinson, Joel D Katz

**Affiliations:** 1 Department of Psychology Faculty of Health York University Toronto, ON Canada

**Keywords:** pain, heuristics, chronic pain, pain inference, numerical anchoring, anchoring effect, mechanical Turk

## Abstract

**Background:**

Numerical anchoring occurs when exposure to a numeric quantity influences a person’s subsequent judgment involving other quantities. This could be applicable to the evaluation of pain, where exposure to an unrelated number before the evaluation of pain could influence pain ratings.

**Objective:**

This study aimed to determine whether exposure to a random numeric anchor influences subsequent pain intensity ratings of a hypothetical patient.

**Methods:**

In this study, 385 participants read a vignette describing a patient with chronic pain before being randomly assigned to one of four groups. Groups 1 and 2 spun an 11-wedge number wheel (0-10), which was, unbeknown to the participants, programmed to stop on a high number (8) or a low number (2), respectively. Group 3 spun a similar letter wheel (A-K), which was programmed to stop on either the letter C or I (control 1). Group 4 did not spin a wheel (control 2). Participants were then asked to rate the patient’s pain intensity using a 0 to 10 numeric rating scale.

**Results:**

The high-number group rated the patient’s pain (median 8, IQR 2) significantly higher than the letter wheel control (median 7, IQR 2; *P*=.02) and the low-number group (median 6, IQR 2; *P*<.001). The low-number group rated the pain significantly lower than controls 1 and 2 (median 7, IQR 2; both *P*=.045).

**Conclusions:**

Pain ratings were influenced by prior exposure to a random number with no relevant information about the patient’s pain, indicating anchoring had occurred. However, contrary to the traditional definition of anchoring where anchoring occurs even when participants are unaware of the anchor’s influence, in this study, the anchoring effect was seen only in participants who believed that the anchor had influenced them. This suggests that anchoring effects could potentially occur among health care providers tasked with evaluating a patient’s pain and should be evaluated further.

## Introduction

### Background

Health care providers are often required to assess and treat pain; however, it is recognized that health care provider ratings of a patient’s pain intensity may be biased and inaccurate [1]. Patients, health care providers, and environmental or situational factors contribute to the providers’ perception and interpretation of a patient’s pain intensity. Examples of factors that have been shown to be associated with biased provider ratings include past work experience [2], physician gender [3], and availability of medical evidence [4]. In these circumstances, provider ratings often do not align with patient ratings and instead tend to over- or under-estimate the patient’s self-report [2,4,5]. It is, therefore, important to consider the processes by which situational factors contribute to biased provider ratings [6].

### Numerical Anchoring

One rarely studied situational factor that appears to contribute to biased health care provider ratings of a patient’s pain intensity has been termed *numerical anchoring*. Numerical anchoring reflects a cognitive bias in which prior exposure to a numeric value influences subsequent numerical decisions. For example, Tversky and Kahneman [7] asked participants to estimate the number of African countries in the United Nations before they spun a rigged wheel with numbers between 0 and 100. The wheel was designed to stop at the number 10 or 65. Participants who spun the wheel that stopped on the number 10 estimated that there were 25 African countries in the United Nations, whereas those who spun the wheel that stopped on the number 65 estimated that there were 45 African countries in the United Nations. Thus, exposure to a prior number anchored participants to a lower or higher value and influenced their response to a later unrelated question. It should be noted that what makes these results especially interesting is that given the situational context of a *random* spinning wheel, the numeric anchor was totally unrelated to the estimation task. The anchor could not possibly provide any useful information about the estimation task, and yet it clearly influenced the participants’ responses. Many studies have since replicated the findings of Tversky and Kahneman [7], namely, that exposure to higher numeric anchors is associated with higher numeric values in subsequent ratings, whereas exposure to lower numeric anchors is associated with lower numeric values [8-11].

Not all anchors are unrelated to the subsequent decision-making process. Anchoring effects have also been studied in relation to pain but to a much lesser extent. Riva et al [6] demonstrated that there may be an anchoring bias in health care professionals’ perceptions of the patient’s pain. The researchers recruited 423 health care professionals who read vignettes describing a patient presenting with a headache. Participants randomized to the experimental arm were asked to rate the patient’s level of pain immediately after reading the vignette and again after learning of the patient’s pain rating, whereas control group participants were asked to rate the patient’s level of pain only after learning of the patient’s self-reported pain level. Health care professionals in the experimental condition tended to maintain their original pain rating or did not sufficiently alter it after hearing the patient’s subjective pain rating. In contrast, those in the control condition tended to agree with the patient’s subjective pain rating. The results of the experimental condition indicate that on the one hand, once an initial judgment of pain had been made by the health care professionals, the patient’s self-reported pain rating did not influence the professionals’ final decision of the patient’s pain intensity [6]. On the other hand, under the appropriate groups, the presence of a pain-related numeric anchor in the form of a patient’s pain rating may unintentionally influence a health care provider’s evaluation of the patient’s pain.

### Objectives

Pain-related numeric anchors appear to influence a health care provider’s perception of the patient’s pain [6]. However, it remains to be seen whether a *random* numeric anchor, with no relevance to the subsequent estimation task, can influence an individual’s perception of someone else’s pain. This has relevance to health care providers, as it would indicate that numeric quantities unrelated to the patient may influence how a health care provider evaluates the patient’s pain. It would also demonstrate a novel situational factor that operates through a cognitive bias to unwittingly influence the health care provider’s estimate of the patient’s pain. This study aimed to provide a preliminary assessment of whether exposure to a *random* numeric anchor influences subsequent estimates of a hypothetical patient’s pain intensity ratings.

### Hypotheses

This study tested four hypotheses. The study’s primary hypothesis (H1) was that participants who were exposed to a random numerical anchor would be influenced by that anchor, with the median pain rating of participants who were exposed to a high numerical anchor being significantly higher than the median pain rating of those who were exposed to a low numerical anchor. The second hypothesis (H2) was that the two groups of control participants who were not exposed to a numerical anchor would not differ in their initial pain intensity ratings. The third hypothesis (H3) was that participants who were originally not exposed to a numerical anchor would instead anchor to their original pain ratings when asked to rerate the patient’s pain, even if they were subsequently exposed to a high anchor. The fourth hypothesis (H4) was that participants who were exposed to a numerical anchor would deny that the anchor influenced their subsequent pain rating and that pain ratings would not differ between those who reported vs those who denied being influenced.

## Methods

### Participants

**Figure 1:**
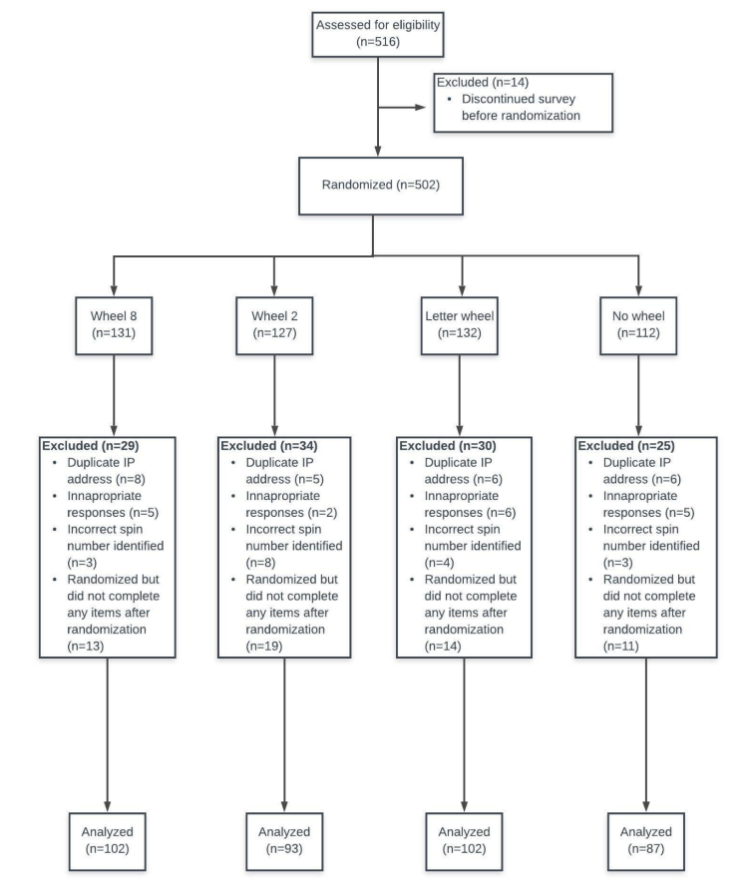
Flow chart of participant recruitment.

A total of 516 participants were recruited through Mechanical Turk (MTurk, version May 2018; Seattle, Washington), a Web-based study recruitment website that has millions of users worldwide who participate in Human Intelligence Tasks in exchange for money [12]. The inclusion criterion was that participants must be fluent in English. Recruitment and survey completion occurred over the period of one day in May of 2018. Of the 516 participants, 385 participants (223 men and 162 women; mean age 35.85 years, SD 10.96; range 19-72 years) were included in the final analysis. Figure 1 shows the flowchart depicting participant recruitment. Participants were excluded from analysis for incorrectly identifying the number that they spun, for duplicate IP addresses, for discontinuing the survey after randomization, or for inappropriate responses to open-ended items. Some examples of inappropriate responses included pasting portions of Wikipedia articles or writing responses unrelated to the questions being asked.

### Procedures

This study was reviewed and approved by the York University Research Ethics Board (Human Participants Review Committee certificate #e2018-017). Participants were recruited through MTurk, where the study was entitled “Answer a psychology survey about pain.” Participants would also see the brief description: “Complete psychological questions and complete a small task on the computer.” MTurk users were compensated US $1 to participate in the study, which took approximately 20 min to complete. We had no restrictions on the location or prior approval rating of the MTurk users. In addition to the survey responses, we also recorded the MTurk user’s internet protocol addresses to eliminate participants who may have attempted to complete the task more than once using multiple MTurk accounts. At the end of the survey, participants received a random code which they subsequently submitted to MTurk to receive payment and confirm that they had completed the survey.

The study was administered using Qualtrics software (version May 2018, Provo, Utah), a Web-based survey management system. Participants were directed to the Qualtrics website, where they first provided informed consent to participate. Each page of the survey consisted of a single question. Participants were unable to return to previous questions after completing an item to maintain the validity of the anchoring process. Participants began the survey by completing demographic questions, including questions regarding their history of pain. We included open-ended questions for participants who endorsed experiencing chronic pain to detail their pain history, which also served as an internal validation question to ensure consistency in participants’ responses. Participants who were inconsistent in their responses were removed from the analysis (“inappropriate responses” in Figure 1). Following demographic and pain items, the participants were then randomized into one of four groups using the block randomizer available in Qualtrics. Each group was asked to read the following vignette, which describes the journey of a hypothetical person from injury, postinjury chronic pain, to rehabilitation:

Steve lives in a modest house on a quiet, tree-lined street very close to a major highway. Last year, as Steve was driving to work one morning, he was involved in a serious collision that nearly cost him his life. He spent months in the hospital and underwent multiple surgeries to repair his leg which was shattered in the crash. After many more months of physical rehabilitation, Steve is left with chronic leg pain and requires a cane to walk especially when the pain acts up. Steve sees his physical therapist once a week for treatment and despite the increased pain he has after each session, he feels the therapy is helping.

**Figure 2:**
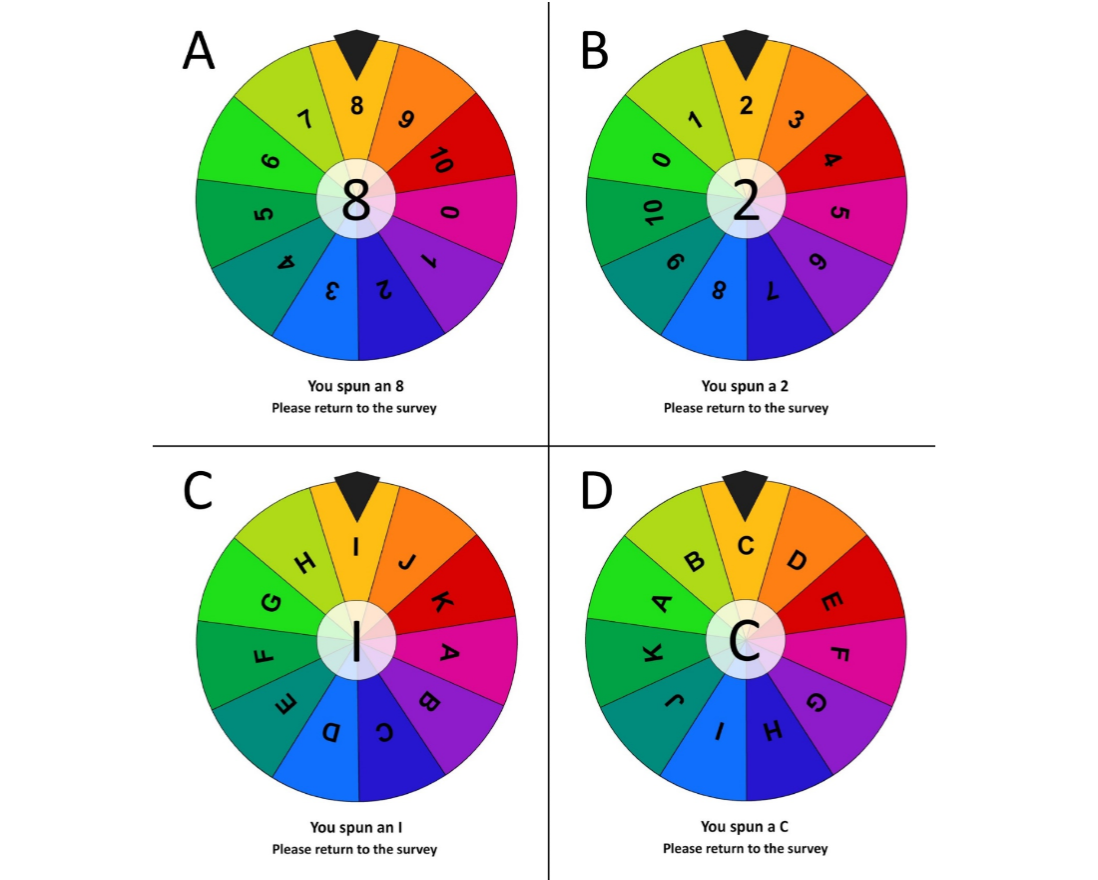
Spinning wheels used for group 1 (A), group 2 (B), and group 3 (C and D).

Virtual spinning wheels comprised 11 wedges, were each created using Adobe Flash (version 2018, Adobe, Seattle, Washington) animation for the purposes of this study. Unbeknown to the participants, these virtual spinning wheels were programmed online to stop at a predetermined value. Participants in group 1 (n=102) and group 2 (n=93) spun a virtual wheel containing the numbers 0 to 10, which was programmed to stop on either a high number (8) or a low number (2), respectively. To control for viewing numeric values, participants in group 3 (n*=*102) spun a similar wheel containing the letters A to K, which was programmed to stop on either the letter *C* or *I*. To control for the spinning of the wheel itself, participants in group 4 (n=87) read the vignette and initially did not spin a wheel. Figure 2 illustrates the wheels used for groups 1, 2, and 3.

Immediately after spinning the wheel, participants in groups 1 and 2 were asked to recall the number they saw on the wheel and to indicate if they thought the number was higher, lower, or equal to the intensity of pain that the patient in the vignette experiences on a typical day. Participants in group 3 were only asked to recall the letter they saw on the wheel spin. Participants in groups 1, 2, and 3 were then asked to estimate the patient’s pain intensity on a typical day using a numeric rating scale (NRS), ranging from 0 (*no pain*) to 10 (*worst possible pain*). Subsequent questions were completed to ascertain whether participants in groups 1, 2, and 3 believed that the anchor had influenced their pain intensity rating of the patient, and if so, in what way. This portion of the survey included a multiple-choice question regarding their belief, with a subsequent open-ended question asking participants to explain why they believed that the anchor did or did not influence their response. Throughout the survey, participants were not given a chance to return to previous questions to maintain the validity of the anchoring process. Multimedia Appendix 1 outlines the full list of anchoring questions that participants were asked.

Participants in group 4 were asked to provide an NRS pain rating immediately after reading the vignette. On providing a pain rating, participants in group 4, who initially did not spin a wheel, were asked to reread the vignette, spin the high-anchor wheel (set to stop on the number 8), and rerate the patient’s pain. This was done to determine whether participants in group 4 would anchor to their own original pain rating or if they would be influenced by the numerical anchor.

After completing the experimental task, all participants completed the Pain Catastrophizing Scale (PCS) questionnaire and the Hospital Anxiety and Depression Scale (HADS) questionnaire, as previous studies have indicated that both pain catastrophizing and anxiety or depression can influence pain ratings [13-16].

### Measures

#### Hospital Anxiety and Depression Scale

HADS measures symptoms of anxiety and depression and has been widely used among both clinical and nonclinical populations [17]. It contains 14 items, consisting of two subscales—seven items comprise the anxiety subscale and seven items comprise the depression subscale. Each item is rated on a 0- to 3-point Likert scale. Higher scores are associated with a higher severity of anxiety or depressive symptoms [17]. Subscale scores range from 0 to 21, where scores equal to or below 7 indicate no clinically relevant findings of depression or anxiety (*normal*). Scores between 8 and 10 are suggestive of a possible mood disorder (*borderline abnormal*), and scores between 11 and 21 are suggestive of the probable presence of a mood disorder (*abnormal*) [15]. HADS has been found to be reliable in detecting states of anxiety and depression and their associated severity. It has good internal consistency (alpha=.82) and has been very well validated in a number of settings [17]. The internal consistency of HADS for this study was 0.91.

#### Pain Catastrophizing Scale

PCS measures the extent to which an individual experiences pain-related catastrophic thinking, including how much they think and worry about pain, magnify the amount of pain experienced, and feel helpless toward painful experiences. It consists of 13 items, each rated on a 5-point Likert scale, with scores ranging from 0 to 52. Scores above 30 are considered to be clinically relevant for catastrophizing [14]. Individuals who score higher on PCS also tend to report more intense pain experiences as well as heightened anxiety and depression symptoms [14]. These individuals also tend to use more analgesic medication, have longer hospitalizations, and tend to demonstrate an increase in pain behaviors and pain-related disabilities [14]. PCS has demonstrated good internal consistency (alpha=.87) and has been well validated in both clinical and nonclinical samples [14]. In this study, the internal consistency of PCS was 0.96.

### Sample Size Estimation

Sample size estimation using G*Power (version 3.1.9.4; University of Düsseldorf, Germany) [18] indicated that 400 participants (n=100 per group) are required for an analysis of variance with an alpha of .05, a power of 0.95, and an effect size of 0.25.

### Data Analysis

Data analyses were conducted with a significance level of .05. Chi-square tests of independence were conducted to determine any significant demographic group differences. A Kruskal-Wallis test was used to determine whether the groups differed in age.

H1 was analyzed using a nonparametric Kruskal-Wallis test, as initial screening of the data revealed a non-normal distribution, necessitating a nonparametric approach to data analysis (see the Results section). The medians of the four groups were compared to determine whether the high and low numerical groups (groups 1 and 2) significantly differed and to determine whether the median pain ratings of groups 3 and 4 were higher than the median pain ratings of group 2 and lower than group 1.

H2, stating that the two control groups (groups 3 and 4) would not significantly differ from one another, was analyzed using a Kruskal-Wallis test.

H3, stating that participants in group 4 would anchor to their original pain ratings rather than be influenced by the high numerical anchor, was analyzed using a Friedman test.

H4, stating that the median pain ratings between participants who believed they had been influenced and participants who believed they had not been influenced by the numerical anchor would not differ, was first analyzed using a chi-square test of independence to determine whether the proportion of participants being influenced by the anchor differed by group. A Kruskal-Wallis test was used to determine if pain intensity ratings were significantly different across groups for those participants who reported they had not been influenced by the anchor and those who felt they had been influenced by the anchor.

## Results

### Demographics

Multimedia Appendix 2 shows the demographic variables for the sample of participants in each of the four groups. The majority of participants self-reported their ethnicity to be white (226/385, 58.7%) or South Asian (97/385, 25.2%). The sample was relatively well educated, with 89.4% (344/385) of participants having at least some postsecondary education. Moreover, 62.6% (241/385) of participants endorsed currently experiencing an ongoing pain problem, with 32.2% (124/385) reporting that they had been diagnosed with chronic pain by a physician. Of the 330 participants on whom longitude and latitude was reported, the majority were located in North America (214/330, 64.8%) or India (95/330, 28.8%), with the remaining participants (21/330, 6.4%) being from South America (8/330, 2.4%), Asia (5/330, 1.5%), Europe (6/330, 1.8%), and Africa (2/330, 0.6%).

### Group Characteristics

Chi-square tests of independence did not demonstrate significant differences between groups in gender, ethnicity, education, or pain history (see Multimedia Appendix 2). Chi-square tests also did not show significant between groups differences in the number of participants who scored above or below the clinical cutoff for PCS (*P*=.26) or for HADS in the depression (*P*=.51) or anxiety (*P*=.30) subscales or in self-reported chronic pain (*P*=.92). A Kruskal-Wallis test demonstrated that there was no significant difference in groups for age (*H*_3_=4.779; *P*=.19). Given that this was a pilot study on the effects of random numerical anchoring on pain inferences, no efforts were made to stratify the sample or analysis.

### Hypothesis 1: The Effects of Numerical Anchoring on Pain Scores

Table 1 shows NRS pain intensity ratings for the four groups.

**Figure 3:**
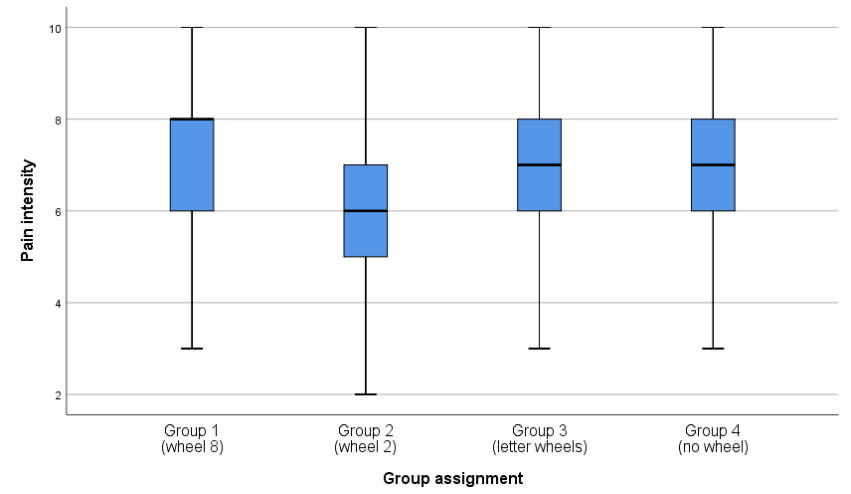
Boxplots of pain intensity ratings for groups 1-4.

A visual inspection of the histograms shown in Multimedia Appendix 3 indicated a non-normal distribution of the pain intensity ratings, particularly for group 1. This was confirmed by the Shapiro-Wilk test (*P*<.001, *P*=.01, *P*=.002, and *P*=.002 for groups 1, 2, 3, and 4, respectively). Table 2 shows the frequency of the pain intensity scores across groups, while Figure 3 shows the box plots of pain scores for the four groups.

**Table 1:** Numeric rating scale pain intensity scores for the four groups.

Pain intensity ratings	Wheel 8 (n=102)	Wheel 2 (n=92)	Letter wheel (n=102)	Control (n=87)
Pain intensity rating, median (IQR)	8 (2)	6 (2)	7 (2)	7 (2)
Pain intensity rating after spinning the wheel (group 4 only), median (IQR)	N/A^a^	N/A	N/A	7 (2)

^a^Not applicable.

**Table 2:** Frequency (percent) of pain intensity ratings for the four groups.

Pain intensity rating (0-10)	Wheel 8 (n=102), n (%)	Wheel 2 (n=92), n (%)	Letter wheel (n=102), n (%)	No wheel (n=87), n (%)
0	0 (0.0)	0 (0)	0 (0.0)	0 (0)
1	0 (0.0)	0 (0)	0 (0.0)	0 (0)
2	0 (0.0)	3 (3)	0 (0.0)	0 (0)
3	2 (2.0)	6 (7)	4 (4.0)	1 (1)
4	6 (6.0)	12 (13)	8 (8.0)	5 (6)
5	15 (15.0)	9 (10)	13 (13.0)	7 (8)
6	12 (12.0)	25 (27)	20 (20.0)	18 (21)
7	12 (12.0)	16 (17)	27 (27.0)	26 (30)
8	40 (39.0)	15 (16)	18 (18.0)	20 (33)
9	9 (9.0)	3 (3)	10 (10.0)	8 (9)
10	6 (6.0)	3 (3)	2 (2.0)	2 (2)

Kruskal-Wallis tests showed a significant difference between the mean ranks of at least one pair of groups in their pain intensity ratings (*H*_3_=19.529; *P*<.001). Dunn pairwise tests revealed that the high-wheel group (median 8, IQR 2) rated the patient’s pain significantly higher than the low-wheel group (median 6, IQR 2; *P<*.001) as well as the letter wheel group (median 7, IQR 2; *P*=.02). There were no significant differences in the pain rating between the high-wheel group and group 4, which initially did not spin a wheel (*P*=.325). The low-wheel group rated the patient’s pain significantly lower than both the letter wheel group (*P*=.045) and group 4, which did not spin a wheel (median 7, IQR 2; *P*=.045).

### Hypothesis 2: Median Pain Ratings of Control Groups

Significant differences were not observed in pain ratings between groups 3 and 4 (*P*=.230).

### Hypothesis 3: Anchoring After an Initial Judgment Had Been Made

A Friedman test indicated that there were no significant differences in pain ratings for group 4 between time 1, initially after reading the vignette (mean_rank_ 1.55), and time 2, after rereading the vignette and spinning the high-anchor wheel (mean_rank_ 1.45; χ^2^_1_=3.2; *P=*.07).

### Hypothesis 4: Influence of the Numerical Anchor

A chi-square test of independence demonstrated that there were significant differences between groups in the proportion of participants who believed that their pain intensity rating of the patient had been influenced by the number they spun (χ^2^_3_=11.0 *P=*.01).

In particular, participants in group 1 were significantly more likely to believe that they had been influenced by the anchor, whereas participants in group 3 were significantly more likely to believe that they had not been influenced by the anchor. In group 1, 35.3% (36/102) of participants endorsed being influenced in comparison with 20% (19/93) of participants in group 2, 16.7% (17/102) of participants in group 3, and 22% (19/87) of participants in group 4 after these participants had spun the high-anchor wheel. Table 3 shows the participants’ perceptions of whether they had been influenced by their group’s corresponding anchor.

**Table 3:** Participants’ perceptions of whether they were influenced by the anchor that they were exposed to.

Influence	Group 1, (n=102), n (%)	Group 2, (n=92), n (%)	Group 3, (n=102), n (%)	Group 4, (n=87), n (%)	Chi-square (*df*)	*P* value
Yes	36 (35.2)	19 (21)	17 (16.7)	19 (22)	11.0 (3)	.01^a^
No	66 (64.7)	74 (80)	85 (83.3)	67 (77)	N/A^b^	N/A

^a^Significance was at an alpha level of .05.

^b^Not applicable.

A Kruskal-Wallis test indicated that among participants who indicated that they had not been influenced by the anchor, there were no significant differences between groups in pain intensity ratings (*H*_3_=7.214; *P*=.07). In contrast, there were significant differences in pain intensity ratings across groups among those participants who indicated they had been influenced by the anchor (*H*_3_=13.644; *P*=.003). Dunn pairwise tests indicated that participants in group 2 (median 6, IQR 5), who spun the low-anchor wheel, rated the patient’s pain significantly lower than participants in group 1 (median 8, IQR 1), who spun the high-anchor wheel (*P*=.003), as well as participants in group 4 (median 8, IQR 2), who initially did not spin a wheel but later spun the high-anchor wheel (*P*=.03). Participants in group 1 who indicated they had been influenced by the anchor reported significantly higher pain intensity ratings than participants in group 3 (median 7, IQR 2), who spun a wheel containing letters (*P*=.006). Finally, among those who believed they had been influenced by the anchor, participants in group 4 rated the patient’s pain significantly higher than participants in group 3 (*P*=.046). In addition, a Kruskal-Wallis test indicated that after participants in group 4 had spun the high-anchor wheel and rerated the patient’s pain, those who indicated that they had been influenced by the anchor tended to rate the patient’s pain as being significantly higher than those who believed they had not been influenced by the anchor (*H*_1_=5.881; *P*=.02). In addition, among those who believed that they had been influenced by the anchor in group 4, there were no significant differences in pain ratings between time 1 (mean_rank_ 1.56) and time 2 (mean_rank_ 1.44; χ^2^_1_ 0.50; *P=*.480).

## Discussion

### Principal Findings

This study examined whether prior exposure to a pain-unrelated, random numerical anchor would influence the participants’ ratings of a hypothetical patient’s pain intensity. This was done by asking participants to read a vignette depicting a hypothetical patient with chronic pain, before asking the participants to spin a wheel, which was programmed to land on a high numerical anchor (8), a low numerical anchor (2), or a letter (C or I). A fourth group served as a control condition and did not spin a wheel initially before rating the patient’s pain intensity but was later asked to spin the high-anchor wheel and rerate the patient’s pain.

The findings supported the main hypothesis in that exposure to a numerical anchor influenced the participants’ estimations of a hypothetical patient’s pain intensity. Participants who spun a high numerical anchor estimated that the hypothetical patient experienced a much higher pain intensity than did the other three groups. In addition, participants in the low numerical anchor condition had the lowest estimation of pain intensity for the hypothetical patient. Importantly, H2 was supported, as there was no difference in pain intensity ratings between participants who spun a wheel containing a letter and the control group that did not spin a wheel, indicating that the spinning of the wheel itself had no effect on pain intensity ratings. These results are in line with studies that have also used a spinning wheel or similar devices to anchor their participants to a random numerical anchor [7,19].

The third hypothesis was supported in that participants who were originally not exposed to an anchor anchored to their original pain rating when asked to rerate the patient’s pain, even when subsequently exposed to the high anchor. Participants did not adjust their second pain rating when asked to rerate the patient’s pain. This was expected, given the results from the study by Riva et al [6], who similarly found that health care providers did not significantly adjust their ratings when asked to rerate a patient’s pain, even when given additional information about the patient’s own subjective pain rating.

The fourth hypothesis, that the median pain ratings in each group would not differ between those who did and did not believe they had been influenced by the anchor, was unsupported. In the high-anchor group, those participants who believed they had been influenced had a significantly higher median pain rating than those who did not believe they had been influenced. Similarly, in the low-anchor group, those participants who believed they had been influenced had a significantly lower median pain rating than those who did not believe they had been influenced. Although the majority of participants in all four groups indicated that they had not been influenced by the anchor, participants who spun a high-anchor wheel were also more likely than any other group to indicate that they had been influenced by the anchor. This may relate to the abovementioned suggestion. The vignette may have depicted a higher pain rating, and after spinning the wheel and rating the patient’s pain as higher, the participants may then have inferred that they must have been influenced. This has been discussed later in detail.

The results also demonstrate that participants who acknowledged the anchor’s influence on their pain rating were, in fact, influenced. Among participants who reported that they had been influenced by the anchor, the results were very similar to the overall study findings in that participants who spun a high-anchor wheel rated the patient’s pain as being more intense than all other groups. In contrast, the median pain ratings for all four groups were not significantly different among those participants who indicated that they had not been influenced by the anchor. In other words, the anchoring effect was seen only in participants who reported being aware of the anchor’s influence on their decision making. These results deviate from previous studies that have examined the role of influence on anchoring effects. Although only one study has looked directly at whether participants believed they had been influenced or not [20], both Chapman and Johnson [21] and Quattrone [22] investigated whether being warned would inhibit the anchoring effect. In all three studies, the anchoring effect was present in all participants who were exposed to the anchor, but anchoring effects were consistently stronger in cases where the anchor was relevant or informative to the target. However, in none of the three studies were the anchors completely random, as they were in this study, despite being uninformative. In the study conducted by Chapman and Johnson [21], participants were anchored to a random dollar amount before being asked target questions about whether they would sell a lottery ticket for that dollar amount. Therefore, although the anchor was random and uninformative, it was not irrelevant to the task at hand. In this study, the anchors used were both irrelevant and random. As such, the results of the previous studies by Chapman and Johnson [21], Wilson et al [20], and Quattrone [22] do not provide a concrete description of the role of influence on random numerical anchoring effects.

The effect of influence that was seen in the three anchoring conditions was also seen in participants in group 4, who initially did not spin a wheel. After rereading the vignette, the participants were asked to spin the high-anchor wheel and rerate the patient’s pain. Overall, the participants did not change their pain intensity rating after rerating the pain, which was expected. Riva et al [6] found similar results in that their participants did not change their pain rating when asked to reevaluate a patient’s pain and instead anchored to their original rating. In this study, however, after spinning the high-anchor wheel, participants in group 4 were also asked whether they believed that the number they spun had influenced their response. Similar to the other three groups in this study, the anchoring effect was seen only in participants who indicated that they had been influenced by the anchor, whereas those who indicated they had not been influenced tended to evaluate the patient’s pain as being less intense and remained consistent with their original pain rating. However, given that the overall participant pain ratings for group 4 did not change between the two time points, it is possible that those who indicated that they had been influenced were the participants who had rated the patient’s pain as being more intense to begin with.

### Interpretations

By taking into consideration the entire sample, the results suggest that anchoring has occurred. However, when considering the effect of influence, anchoring only appears to have occurred in those who reported that they had been influenced. These findings are contradictory to the traditional definition of anchoring, where anchoring is conceived as an implicit cognitive process and is thought to occur regardless of the participant’s awareness of the anchor’s influence on their subsequent decisions.

The effect of influence rarely has been studied in anchoring. Given the traditional anchoring template as designed by Tversky and Kahneman [7], where influence is not explored, the majority of anchoring researchers typically have not included a question aimed at determining the role of influence on participants’ decision making [23]. However, Wilson et al [20] did explore the role of influence on anchoring. In a series of two studies, participants were asked to rate how much they believed their answers had been influenced by the anchor on a 9-point Likert scale. Similar to this study, the majority of their participants believed that the anchor had no influence on their response, and higher anchor values were associated with more belief of the anchor’s influence. However, in both the studies conducted by Wilson et al [20], anchoring effects were found even in those who did not acknowledge the anchor’s influence. We were unable to replicate these results in this study, in that, across groups, the anchoring effect was diminished when participants did not believe that the anchor had influenced them. In other words, the anchoring effect was contingent upon the participants’ acknowledgment that they had been influenced by the anchor.

It is possible that these influence effects seen across groups are because of a confirmatory search mechanism, as proposed by Chapman and Johnson [21], in that, after being exposed to a numerical anchor, participants focus on reasons why that number is consistent with the hypothetical patient’s pain, rather than on reasons why the anchor may be inconsistent with the patient’s pain intensity. In this way, the numerical anchor may have influenced their decision making. It is interesting, however, that participants in group 3 who spun a letter wheel indicated that they had been influenced by the anchor, despite the anchor being a letter value rather than a numeric one and, therefore, holding no possible relevant information for an NRS. This finding may be because of a demand effect [24,25], where participants may have inferred that they would not have been asked to spin a letter or had their attention subsequently drawn to it through the questions asked of them, if the letter was not relevant or informative in some way. Finally, as noted by Nisbett and Ross [26], these influence effects do not necessarily indicate that those who believed that they were influenced, actually were influenced. Rather, it may be that after being exposed to the anchor, the participants inferred that their judgment must have been influenced based on the response that they gave [20].

### Limitations

This study has a number of limitations that are important to consider. Given that the study was completed online, it is possible that participants were not able to fully attend to the vignette, the wheel, or the subsequent questions. As a result, the anchoring effects and influence effects seen may be instead due to the fact that the participants had very recently been exposed to a number rather than the true anchoring effects, ie, if participants were not attending fully, they may have rated the participant’s pain according to the numerical anchor they were exposed to simply because of the availability of the anchor in their memory rather than because that is the pain intensity rating they believe the patient experiences or because of anchoring effects. These same participants might subsequently indicate that they had been influenced by the anchor, as their response was based on the number they had been exposed to. Previous studies have demonstrated that data collected through MTurk are as reliable as data collected in a laboratory setting, with the exception of attention paid to the study itself [27,28]. Typically, this limitation is overcome through the use of validity questions to ensure that the participant is attending the study [27]. This study did contain validity questions, such as asking the participants which number or letter they spun; however, it is possible that additional validity questions regarding the vignette would have helped to more effectively screen out inattention.

A second limitation is that this study has no pilot data on the vignette that was used to give a description of the hypothetical patient. As a result, it is unknown what the patient’s baseline pain intensity would be rated as. This information would help to ensure that the vignette itself was not a confounding variable. For instance, if the vignette was shown to depict a pain intensity that is higher without the presence of a numerical anchor, it is possible that the influence effect that was seen in the high-anchor group may have been because of participants inferring that they had been influenced, given the pain intensity rating that they had given.

Finally, this study is limited by the fact that it is one of the first anchoring studies to look at the effect of influence on anchoring effects. As such, the questions regarding influence had not been previously tested and may not have been valid or may have unwittingly created biased responses.

### Strengths

Despite the abovementioned limitations, the study also has a number of strengths. First, with a relatively large sample size of participants who were recruited globally, it is likely that the data are not only reliable but also cross-culturally validated. Participants were diverse in their age, education, ethnicity, and pain history, which also helps to ensure that the data are valid and generalizable. Although participant characteristics are often unreported in studies that use crowdsourcing such as MTurk [29], these data allow this study’s findings to be more easily replicated and interpreted. To ensure that the results would be generalizable, MTurk was chosen as the primary recruitment method for this study, as previous studies have shown that the data collected through MTurk is as reliable as data gathered from undergraduate participants [30,31] or other laboratory-sourced participants [32].

Second, this study is strengthened by the presence of two control conditions. In this way, both the effect of spinning a wheel and the effect of having the wheel land on a number could be controlled. This helps to ensure that the anchoring effects seen are, in fact, because of anchoring effects, as opposed to being because of a confounding variable.

Finally, this study is one of the only studies to have looked at the effect of influence and found that anchoring effects were contingent upon the participant’s belief that they had been influenced. Anchoring research has been very robust and well established, but there has been very little research on the effect of influence on anchoring and what these findings mean for the definition of anchoring itself. This study’s results may help to better understand anchoring effects as a whole as well as its underlying cognitive pathways.

### Future Directions

Future studies should attempt to clarify the role of influence on numerical anchoring. Namely, attempts should be made to replicate anchoring studies while also considering the participant’s perception of influence. It may be that the current definition of anchoring is not suitable if the effects of influence are reliably seen across studies, given that the current definition implies that participants are not aware of the anchor’s influence on their judgment. Future studies should also expand on this research about how random numerical anchoring might affect the pain response. It would be interesting to determine whether these same random numerical anchors would affect a participant’s judgment of their own pain experience in both acute and chronic pain patients. Future studies may also look at how numerical anchoring may be evident in the health care context in relation to how random numerical anchors may influence a health care provider’s judgment and treatment of a chronic pain patient’s experience.

### Conclusions

The results of this study are consistent with previous studies of numerical anchoring. Exposure to a high numerical anchor influenced participants’ subsequent rating of a hypothetical patient’s pain to be higher, whereas exposure to a low numerical anchor influenced participants to rate the patient’s pain as lower. However, although the majority of participants across groups did not believe they were influenced by the anchor, the anchoring effect was seen only in participants who did indicate that the anchor had influenced their judgments. Further research is necessary to determine the role of influence on anchoring effects and the applicability of anchoring effects in the health care context.

## Appendix

Multimedia Appendix 1 Anchoring questions.

Multimedia Appendix 2 Demographic information for the four groups.

Multimedia Appendix 3 Frequency distributions of pain intensity ratings for groups 1-4.

## Abbreviations

HADS: Hospital Anxiety and Depression Scale
MTurk: Mechanical Turk
NRS: numeric rating scale
PCS: Pain Catastrophizing Scale
