# Supplementary material for: Influencing Pain Inferences Using Random Numerical Anchoring: Randomized Controlled Trial
Source: JMIR Hum Factors. 2020 Mar 9;7(1):e17533. doi: 10.2196/17533 (PMC7091028; doi:10.2196/17533)
Supplement: Multimedia Appendix 1 [file humanfactors_v7i1e17533_app1.docx]

**Multimedia Appendix 2**

*Anchoring Questions*

1. What number/letter did you spin*?
2. Using the above scale, do you think Steve’s pain intensity on a typical day is higher than, lower than, or equal to the number you just spun? **/***
   1. Higher
   2. Lower
   3. Equal
3. Using the 0-10 scale above, how intense do you think Steve’s pain is on a typical day?
   1. Why did you choose that number?
4. Do you think that the number/letter you spun influenced your rating of Steve’s pain? */***
   1. Why do you think the number/letter influenced your rating of Steve’s pain?
   2. Why do you think the number/letter you spun did not influence your rating of Steve’s pain?
5. If a health-care professional were to rate Steve’s pain, how intense do you think they would rate it to be?
   1. What made you choose that number?
6. Please re-read the vignette.

Steve lives in a modest house on a quiet, tree-lined street very close to a major highway. Last year, as Steve was driving to work one morning, he was involved in a serious collision that nearly cost him his life. He spent months in the hospital and underwent multiple surgeries to repair his leg which was shattered in the crash. After many more months of physical rehabilitation, Steve is left with chronic leg pain and requires a cane to walk especially when the pain acts up. Steve sees his physical therapist once a week for treatment and despite the increased pain he has after each session, he feels the therapy is helping.

Now imagine you hadn’t spun the wheel.  Please use the 0-10 scale shown above to rate how intense you think Steve’s pain is on a typical day.

**** This item was customized to say “number” or “letter” depending on group assignment.**

**** indicates an item that was asked only for Groups 1 and 2**

***** Indicates an item that was not asked for Group 4**
