# Supplementary material for: Influencing Pain Inferences Using Random Numerical Anchoring: Randomized Controlled Trial
Source: JMIR Hum Factors. 2020 Mar 9;7(1):e17533. doi: 10.2196/17533 (PMC7091028; doi:10.2196/17533)
Supplement: Multimedia Appendix 2 [file humanfactors_v7i1e17533_app2.docx]

**Multimedia Appendix 1**

Demographic information for the four groups

|  |  | **Group 1**  **(*n=* 102)** | **Group 2**  ***(n*= 92)** | **Group 3**  **(*n*= 102)** | **Group 4**  **(*n*= 87)** | **X(df)** | ***p*** |
| --- | --- | --- | --- | --- | --- | --- | --- |
| **Age (years) (SD)** | | 35.06 (10.11) | 34.80 (9.47) | 37.13 (11.63) | 39.34 (11.84) |  |  |
| **Sex *n* (%)** | | | | | |  |  |
|  | Male | 64 (62.7%) | 53 (57%) | 50 (49%) | 55 (63.2%) | 5.319(3) | .150 |
|  | Female | 38 (37.3%) | 40 (43%) | 52 (51%) | 32 (36.8%) |  |  |
| **Education *n* (%)** | | | | | |  |  |
|  | High School | 4 (3.9%) | 12 (12.9%) | 15 (14.7%) | 10 (11.5%) | 16.456(9) | .058 |
|  | Some post-secondary | 33 (32.4%) | 18 (19.4%) | 17 (16.7%) | 22 (25.3%) |  |  |
|  | Post-secondary | 41 (40.2%) | 49 (52.7%) | 47 (46.1%) | 38 (43.7%) |  |  |
|  | Graduate degree | 24 (23.5%) | 14 (15.1%) | 23 (22.5%) | 17 (19.5%) |  |  |
| **Ethnicity *n* (%)** | | | | | |  |  |
|  | White | 62 (60.8%) | 51 (54.8%) | 62 (60.8%) | 51 (58.6%) | 9.044(12) | .699 |
|  | South Asian | 23 (22.5%) | 27 (29%) | 26 (25.5%) | 21 (24.1%) |  |  |
|  | African Descent | 5 (4.9%) | 5 (5.4%) | 6 (5.9%) | 10 (11.5%) |  |  |
|  | Other | 11 (10.8%) | 10 (10.8%) | 7 (6.9%) | 5 (5.7%) |  |  |
| **Ongoing Pain *n* (%)** | | | | | | | |
|  | Yes | 63 (61.8%) | 56 (60.2%) | 72 (70.6%) | 49 (56.3%) | 8.030(6) | .236 |
|  | No | 39 (38.2%) | 35 (37.6%) | 30 (29.4%) | 37 (42.5%) |  |  |
| **Pain Duration *n* (%)** | | | | | | | |
|  | < 3 months | 9 (8.8%) | 5 (5.4%) | 10 (9.8%) | 6 (6.9%) | 12.869(12) | .379 |
|  | 3-6 months | 6 (5.9%) | 12 (12.9%) | 14 (13.7%) | 2 (2.3%) |  |  |
|  | 6-12 months | 9 (8.8%) | 10 (10.8%) | 8 (7.8%) | 6 (6.9%) |  |  |
|  | >1 year | 38 (37.3%) | 30 (32.3%) | 40 (39.2%) | 35 (40.2%) |  |  |
| **Pain longer than 3 months *n* (%)** | | | | | | | |
|  | Yes | 39 (38.2%) | 42 (45.2%) | 32 (31.4%) | 38 (43.7%) | 5.989(6) | .424 |
|  | No | 63 (61.8%) | 50 (53.8%) | 69 (67.6%) | 48 (55.2%) |  |  |
| **Chronic Pain *n* (%)** | | | | | |  |  |
|  | Yes | 29 (28.4%) | 32 (34.4%) | 33 (32.4%) | 29 (33.3%) | 2.023(6) | .918 |
|  | No | 72 (70.6%) | 59 (63.4%) | 68 (66.7%) | 56 (64.4%) |  |  |
| **Classification of HADS Depression Scores *n* (%)** | | | | | | | |
|  | Normal | 70 (68.6%) | 65 (69.9%) | 66 (64.7%) | 53 (60.9%) | 5.679(6) | .450 |
|  | Borderline | 25 (24.5%) | 21 (22.6%) | 31 (30.4%) | 31 (35.6%) |  |  |
|  | Abnormal | 7 (6.9%) | 7 (7.5%) | 5 (4.9%) | 3 (3.4%) |  |  |
| **Classification of HADS Anxiety Scores *n* (%)** | | | | | | | |
|  | Normal | 48 (47.1%) | 48 (51.6%) | 49 (48%) | 55 (63.2%) | 7.755(6) | .257 |
|  | Borderline | 24 (23.5%) | 17 (18.3%) | 26 (25.5%) | 12 (13.8%) |  |  |
|  | Abnormal | 30 (29.4%) | 28 (30.1%) | 27 (26.5%) | 20 (23%) |  |  |

*Note.* * indicates significance at the α=.05 level. *SD*= Standard Deviation
