# Supplementary figures and images for: Influencing Pain Inferences Using Random Numerical Anchoring: Randomized Controlled Trial
Source: JMIR Hum Factors. 2020 Mar 9;7(1):e17533. doi: 10.2196/17533 (PMC7091028; doi:10.2196/17533)

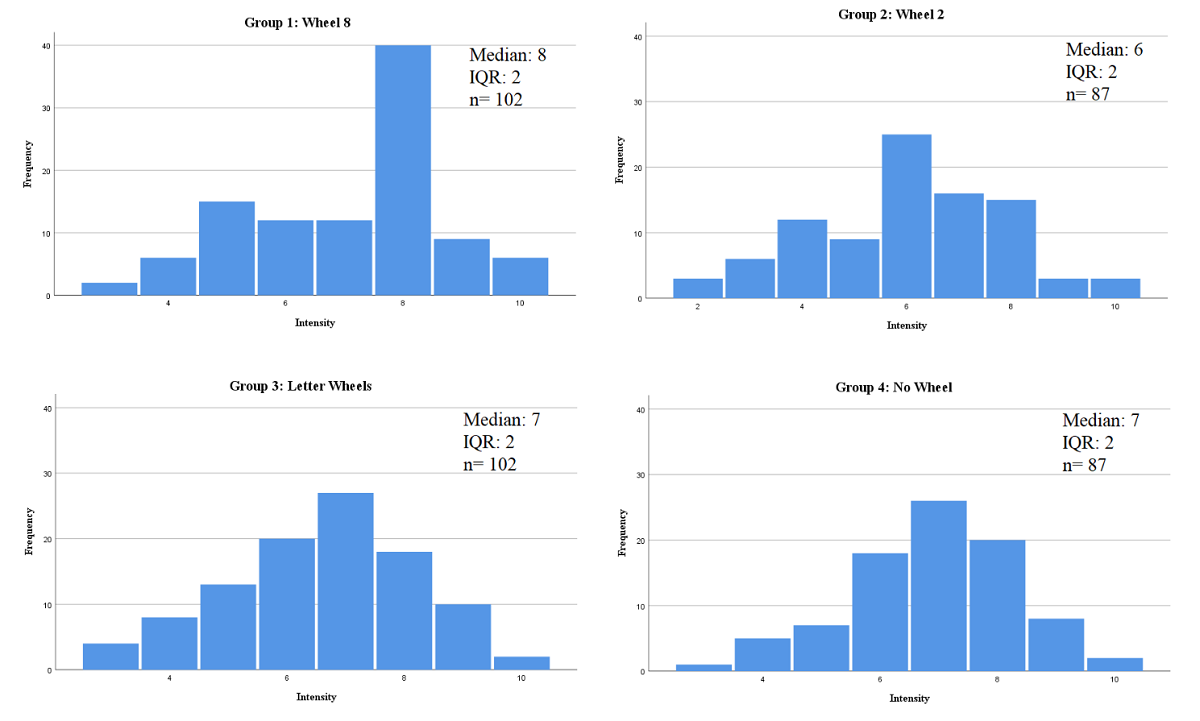

Supplement: Multimedia Appendix 3 [file humanfactors_v7i1e17533_app3.png]
